# Perceived Emotional Self-Efficacy and Life Satisfaction of Elementary School Children on the US-Mexico Border

**DOI:** 10.1177/2333794X241286719

**Published:** 2024-09-27

**Authors:** Mei-Ling Lin, Yok-Fong Paat

**Affiliations:** 1 The University of Texas Health Science Center at San Antonio, San Antonio, TX, USA; 2 The University of Texas at El Paso, El Paso, TX, USA

**Keywords:** elementary school students, emotional self-efficacy, life satisfaction, Hispanic origin students, gender differences

## Abstract

*Objectives.* It has been established that an adult’s perceived ability to effectively address negative emotions predicts their life satisfaction. To increase the understanding of ethnic minority children’s mental health and quality of life, this study examined the relationship between perceived emotional self-efficacy and life satisfaction of Hispanic children. *Methods.* Using the nonexperimental–correlational research design and the convenience sampling method, a total of 176 fourth-, fifth-, and sixth-grade students (73 boys, 103 girls; 88% Hispanic) in one public elementary school on the US-Mexico border were recruited to participate in this study. Emotional self-efficacy was measured using the emotional subscale of the Self-Efficacy Questionnaire for Children and subjective well-being was measured using the Student Life Satisfaction Scale. Spearman correlation and ordinal regression analyses were used to test the study hypotheses. *Results.* Consistent with findings from the current literature, emotional self-efficacy was positively associated with subjective well-being. Children in lower elementary grades were more likely to report higher emotional self-efficacy than those in higher elementary grades. Boys were more likely to report higher life satisfaction than girls. *Conclusions and Relevance.* Using a sample of elementary school children with Hispanic backgrounds on the US-Mexico border, this study attested to the empirical link between emotional self-efficacy and life satisfaction. Our study findings stress the importance of early identification of students with low emotional self-efficacious beliefs and the early introduction of social-emotional learning programs in elementary schools to enhance students’ emotional self-efficacy. This study contributes to positive psychology literature and provides insights for future school-based mental health practice and research.

## Introduction

Middle childhood is a critical development period characterized by rapid growth milestones (emotional, social, physical, and cognitive).^1^ Identification of factors that contribute to their emotional and mental well-being can help support positive outcomes across the lifespan including problem-solving abilities, and emotional regulation. Enhancing children with skills and resources to bolster their overall well-being can help them build resilience in the face of adversity.^2^ Among all, emotional self-efficacy and life satisfaction are two important assets that play a pivotal role as they navigate their development phases and later life paths.^3^

### Emotional Self-Efficacy

Children’s emotional self-efficacy (ESE) is their perceived ability to react to, manage, and regulate emotions, both negative (e.g., anxiety, fear, and depression) and positive (e.g., happiness and confidence).^4,5^ According to Bandura’s self-efficacy model, children develop ESE by integrating information derived from personal accomplishments in the effective management of stressful events, one’s performance in comparison with other peers’ or adults’ performance during stressful events, positive or negative feedback about their socioemotional capabilities, and overall health status. All of these suggest the importance of adults’ involvement in the development of children’s ESE. This study focused on children’s ESE specifically as it relates to managing negative emotions.

Emotional self-efficacy serves as a psychological asset that protects children and youth from undesirable developmental outcomes and contributes to their mental health, general well-being,^4,6^ and life satisfaction.^7,8^ Using samples of middle and high school students, recent literature has provided considerable evidence of significant negative correlations between ESE and internalizing behaviors,^8,9^ depressive symptoms,^10-12^ anxiety and panic disorders,^13,14^ externalizing behaviors,^8,9^ abnormal eating behaviors,^15^ bullying and victimization,^16^ and suicide ideation and attempts.^17^ Additionally, longitudinal investigations of ESE support that lower ESE predicts more behavioral problems in later stages of development.^18^ In contrast, higher ESE has been shown to predict better social adaptation^19^ and academic achievement^18,20^ following the transition from primary to secondary school. These findings provide valuable insights for interventionists to address ESE issues in programs that target youth in transition, but not younger children. Understanding and fostering the emotional self-efficacy of children is imperative in addressing the mental health issues (e.g., anxieties, social withdraws, and conduct and behavioral problems) encountered by this age group.^21^

The Self-Efficacy Questionnaire for Children (SEQ-C)^12^ is the most commonly used ESE measure in empirical studies of children and youth. Originally developed in Belgium, the SEQ-C comprises academic, emotional, and social self-efficacy domains. Empirical evidence about the relationship between gender and ESE is mixed. For example, Suldo and Shaffer^8^ discovered that, among 697 US students in the seventh through 12th grades (58% African American and 36% Caucasian), boys reported significantly higher ESE than girls. Conversely, researchers in India^22^ and Spain^23^ found no statistically significant differences between male and female middle and high school students regarding ESE. Nevertheless, few studies have been undertaken to better understand how this plays out among Hispanic children. The current study examined gender differences in ESE and addressed the significant under-representation of Hispanic children and youth in ESE studies by including a predominantly Hispanic children sample.^8,22,23^

### Life Satisfaction

Life satisfaction, which is conceptualized as a subjective evaluation of one’s life circumstances and the fulfillment of personal goals and desires, is considered a component of one’s overall subjective well-being (SWB).^24,25^ Notably in literature, the study of life satisfaction in adults has received considerable attention. The empirical findings from various life satisfaction measures consistently highlight the importance of the life satisfaction construct in relation to mental health^26^ and as a targeted outcome for numerous psychotherapy interventions.^27^ Compared to the extensive study of life satisfaction in adults, the study of life satisfaction in elementary school children is relatively less examined but has gained more empirical attention recently^28^; this is partially due to the limited availability of reliable and valid measures for this age range, considering their cognitive and emotional developmental stages.^24,29^

The Students’ Life Satisfaction Scale (SLSS)^24^ is one of the few available life satisfaction measures for children in Grades 3 to 12. Since its initial development, the SLSS has been heavily used in pediatric populations from different countries.^30-33^ Results from an earlier correlational study revealed that students who indicated a higher level of life satisfaction tended to rate their self-esteem, internal locus of control, and extraversion measures higher while measures of anxiety and neuroticism lower.^34^ Using a sample of 779 students (53% female, 62% Caucasian) from a Southeastern US middle school district, Lewis et al.^35^ found a positive relationship between life satisfaction and cognitive engagement (i.e., self-regulation, understanding the importance of school, students’ investment in learning, and desire for challenge).

Contrary to Huebner’s discoveries that children’s ratings on SLSS were not significantly influenced by such demographic variables as age, gender, grade, parents’ marital status, or parent’s occupational status,^34^ Aymerich and Casas,^36^ which explored the use of the Present Overall Life Satisfaction (POLS) in a sample of 614 adolescents (age range = 16-19), found that the life satisfaction scores were higher among boys than among girls. These significantly lower levels of satisfaction in adolescent girls were also associated with higher levels of anxiety and depression than among boys. Findings from subsequent SLSS studies showed that satisfaction with parent-child relationships or family life was the strongest predictor of elementary schoolers’ perceived life satisfaction.^37^ Further, using a nationally representative sample of 3358 middle school students from Norway, Danielsen et al. noted that general self-efficacious beliefs significantly predicted life satisfaction.^38^

Recent studies that utilized the SLSS have reported that perceived life satisfaction among children and youth has important implications for their psychosocial and academic functioning. Limited studies, however, are exploring the relationship between emotional self-efficacious beliefs and life satisfaction.^38^ Also, study participants in SLSS research to date have been composed predominantly of Caucasian students.^39^ While preliminary evidence suggests that the SLSS did not display differential psychometric properties based on a child’s race, conducting studies that recruit children from diverse ethnic groups would significantly contribute to the understanding of life satisfaction across different populations.

### The Current Study

To address the knowledge gap in current literature, this study examined the relationships between ESE and life satisfaction among elementary-aged students with predominantly Hispanic origins. It is important to note that in the elementary school where data were collected for this study, parents could decide whether or not to keep their children in the same elementary school through the sixth grade. In other words, sixth grade is included in this particular elementary school whereas sixth grade typically is housed in middle schools in the US. As part of a larger intervention research project, this study aimed to accomplish the following objectives:

(1) Examine the relationship between perceived ESE and life satisfaction from the perspectives of elementary school children and how gender and grade level moderated this relationship. Based on the positive correlations between ESE and health and academic outcomes found in the literature,^18-20^ a statistically significant positive prediction of ESE on life satisfaction was hypothesized.
(2) Understand the relationship between gender and grade level with ESE. The male gender was hypothesized to be a statistically significant predictor for emotional self-efficacy based on an early ESE study conducted in the US.^8^
(3) Determine the relationship between gender and grade level with life satisfaction. It was hypothesized that the perceived life satisfaction ratings were not significantly influenced by gender and grade level, based on Huebner.^34^

## Method

### Study Design

This study adopted a nonexperimental–correlational research design to examine the relationship between ESE and life satisfaction and the roles of grade level and gender play in this relationship.

### Setting and Participants

Data were collected from one elementary school located in one US-Mexico border community. Of the 549 students attending the school at the time of the study, 71% were from households that have been economically/socially marginalized, 88% were from Hispanic origins, and 26% were English language learners. Using convenience sampling, all fourth through sixth graders (N = 249) in general education classrooms who were able to comprehend the survey questions and response options either through reading or listening to English/Spanish were recruited to participate in this study at the beginning of the fall semester of 2019. Also, all fourth and fifth graders (except for transfer students and students who were absent on the day of the program’s implementation) received a 45-minute mindfulness-based program in the spring semester of 2019 (Lin, 2020).^40^

A total of 176 children (73 boys and 103 girls) participated in this study, with 43 in sixth grade, 57 in fifth grade, and 76 in fourth grade. Grade levels instead of chronological ages of students were selected for data analysis in this study because grade levels offer a more accurate representation of participants’ educational development and readiness for answering item questions in the SLSS. After signing the assent forms, participants were asked to complete 2 questionnaires in their designated space in the classroom. The study took place between September to December 2019. The school counselor, nurse, and authors were available to assist children if needed and to ensure confidential and independent responses. Consistency among the three was maintained through regular communication, coordination, and adherence to ethical guidelines, ensuring a cohesive and reliable approach to assisting the children and collecting data.

### Variables and Measurements

Children’s ESE was measured using their total ratings on the emotional subscale of the Self-Efficacy Questionnaire for Children (SEQ-C). The SEQ-C is known to demonstrate strong internal consistency, construct validity,^8,11^ and cross-ethnicity validity.^41^ A total of eight items were validated to measure the subject’s perceived capability to cope with negative emotions^12^ Using a 5-point scale ranging from “not at all” (1) to “very well” (5). For this study, two words were added to two of the item questions: “How well can you prevent yourself from becoming *too* nervous” and “How well can you control your *negative* feelings.” Internal consistency estimates for the SEQ-C results in this study were acceptable (α = .69). Ongoing monitoring and evaluation of data collection procedures were carried out to maintain the quality and consistency of the participants’ responses. The word “too” was added because the research team believed that being nervous is not necessary a negative feeling, rather a common response many people encounter on a daily basis. In addition, since this study focused on children’s ESE specifically as it relates to managing negative emotions, the word “negative” was added.

The participants’ subjective well-being was measured using their total ratings on the Student Life Satisfaction Scale (SLSS),^24^ which is a brief seven-item measure of life satisfaction designed for use with children as young as eight years old and across a wide range of intellectual abilities. The SLSS demonstrates acceptable internal consistency and correlates with criterion measures that are based on predominantly Caucasian samples.^24,33-35,42,43^ Participants were required to rate their well-being during the past several weeks using a 4-point scale that consists of “never,” “sometimes,” “often,” and “almost always” responses. Two item scores in the SLSS were reversed when calculating the SLSS total scores. Internal consistency estimates for the SLSS in this study were considered good (α = .82). Child’s gender (male vs female) and grade level (fourth, fifth, and sixth grades) were used for the correlation and regression models.

Since both assessments used in this study are freely available and do not have copyright restrictions, no permissions were required from any copyright holder.

### Data Management and Analysis

The Statistical Package for Social Sciences (SPSS v. 26) was used to assist in data management and analysis procedures. First, data distribution and missing data patterns between gender groups and across grade-level groups were examined using descriptive statistics. To examine the correlations between ESE and life satisfaction, Spearman correlation coefficients were computed for the full sample and gender and grade-level subsamples, given the ordinal variables used in both measures. Ordinal regression models were used to investigate whether age and grade level are significant predictors for ESE and life satisfaction.

### Ethical Approval and Informed Consent

This study was approved by the University Research Ethics Committee (approval no. 1362238-1) on March 28, 2019 and the School District Assessment, Research, Evaluation and Accountability Office (approval no. 679) on August 8, 2019. All participants provided written informed consent prior to enrollment in the study. Written informed consent to participate in this study was provided by the participants’ legal guardians/next of kin.

## Results

### Descriptive Findings

Missing values were less than 5% of the total number of cases and were considered missing completely at random. Thus, listwise deletion was used. Table 1 shows descriptive statistics for ESE and life satisfaction according to the two grouping variables, gender and grade level. Boys reported higher SEQ-C and SLSS scores than girls, fourth graders reported the highest SEQ-C scores, and students across the three grade levels reported comparable SLSS scores. Distributions of life satisfaction ratings for boys and sixth graders were highly skewed.

**Table 1.** Descriptive Statistics of Study Variables.

Variable	n	M	SD	Range	Minimum	Maximum	Skewness
SEQ-C	170	23.61	6.37	32	8	40	−.02
Male	70	24.54	6.67	32	8	40	−.21
Female	99	23.00	6.11	27	11	38	.07
Fourth Grade	70	24.59	6.56	32	8	40	−.05
Fifth Grade	57	23.96	5.68	26	8	34	−.10
Sixth Grade	43	21.53	6.56	26	8	34	.18
SLSS	169	30.02	7.81	34	8	42	−.83
Male	69	31.49	7.46	34	8	42	−1.08
Female	99	28.96	7.95	33	8	41	−.68
Fourth Grade	73	29.85	8.16	31	11	42	−.51
Fifth Grade	54	29.91	7.91	34	8	42	−.99
Sixth Grade	42	30.48	7.21	31	8	39	−1.37

### Correlations Between Emotional Self-Efficacy and Life Satisfaction

Table 2 shows the correlations between SEQ-C and SLSS scores for the full sample and subgroups. The Spearman correlation coefficients were all statistically significant. A stronger linear relationship was evident between SEQ-C and SLSS scores for boys and fourth graders compared to the other groups.

**Table 2.** Correlations Between SEQ-C and SLSS Scores.

	Life Satisfaction (SLSS)					
	Full Sample	Boys	Girls	Fourth grade	Fifth grade	Sixth grade
Emotional self-efficacy (SEQ-C)	.46**	.53**	.41**	.56**	.44**	.37**

** *P* < .01, two-tailed.

### Predictors of Emotional Self-Efficacy and Life Satisfaction

Two assumption tests were performed prior to ordinal regression analysis. The results from the multicollinearity and full likelihood ratio tests indicated no multicollinearity between the gender and grade-level variables. In addition, the gender and grade-level variables each had an identical effect at each cumulative split of the ESE and life satisfaction variables. Moreover, both the Pearson chi-square test results [χ^2^(133) = 138.48, *P* = .36 for SEQC-E; χ^2^(158) = 164.25, *P* = .35 for SLSS] and deviance test results [χ^2^(133) = 149.59, *P* = .15 for SEQ-C-E; χ^2^(158) = 168.70, *P* = .27 for SLSS] were non-significant, suggesting good model fits.

The model results showed that grade level is a statistically significant predictor for perceived emotional self-efficacy. The odds of sixth graders reporting low SEQC-E scores were 0.395 (95% CI, 0.728-0.061) times that of fourth graders, which was a statistically significant effect (Wald χ^2^(1) = 5.389, *P* = .022). Notably, gender was a significant predictor for perceived life satisfaction. The odds of girls reporting low SLSS scores were 0.585 (95% CI, 0.045-1.124) times that of boys, which was a statistically significant effect (Wald χ^2^(1) = 4.512, *P* = .034).

## Discussion

### The Relationship Between Emotional Self-Efficacy and Life Satisfaction and How Gender and Grade Level Moderated This Relationship

This study found that higher ESE was associated with higher levels of life satisfaction in a sample of elementary school children. The statistically significant correlations were consistent across both genders and three grade levels, with boys and fourth graders demonstrating the strongest linear relationship. In other studies using youth samples, positive correlations among ESE, life satisfaction,^7,8^ and mental well-being^6^ are consistent. Notably, ESE is negatively correlated with psychopathological symptoms^8,9,17^ and less favorable developmental outcomes.^15,16^ This study’s findings contribute to positive psychology literature and support the criterion validity of the SEQ-C through its application in a sample of predominantly Hispanic children as young as those in fourth grade.

Furthermore, the mean emotional domain’s total SEQ-C score of 23.61 found in this study is higher than those found in previous youth studies. In general, general high schoolers perceive higher ESE (M = 22.58^8^; M = 27.46^22^) than general middle schoolers (M = 18.8^16^; M = 20.57^4^) and adolescents in institutional care (M = 22.01^11^). One potential explanation for this result could be that elementary school students face relatively simpler academic challenges and less complex interpersonal issues compared to older students. Consequently, they may tend to overestimate their emotional self-efficacy abilities. Bandura’s self-efficacy model^5^ highlights the importance of positive experiences of stress management and social-emotional support from adult and peer role models to enhance self-efficacy development. Yet, given these factors were not explored in the current study, future longitudinal research endeavor to examine this relationship is warranted, especially in terms of how it develops during early school years and the differential social impacts on its formation across developmental stages.

The mean total SLSS score of 30.02 obtained in this study is similar to that found in the literature that examined elementary schoolchildren samples.^29^ Both the SEQ-C and SLSS should be reassessed periodically for students within a given school to create local norms for schools and subgroups (e.g., grade levels). Furthermore, meta-analyses of multiple studies of elementary school children would be beneficial to determine typical average levels for ESE and life satisfaction. School mental health providers can use these data to target children who show early signs of emotional and behavioral difficulties and intervene promptly to mitigate mental health disorders and illnesses.^44^

### The Relationship Between Gender and Grade Level With Emotional Self-Efficacy

This study found that students in the higher elementary grades were more likely to report lower ESE scores than younger elementary school children. As such, we cannot assume that elementary school children’s confidence and competence in managing negative emotions will grow and improve with age. One possible explanation for this finding is that older students are experiencing greater academic, interpersonal, and emotional stress than their younger counterparts.^45^ Also, the mindfulness-based program that all the fourth and fifth graders received prior to this study might have contributed to the accumulation of successful experiences in handling stress and anxiety and thus elicited positive impacts on reported ESE. Considering this potential confounding factor, caution should be exercised when interpreting this finding. Furthermore, additional studies should be conducted to determine if similar results can be replicated.

Although the average SEQ-C scores were higher for boys than girls in this study, gender was not a statistically significant predictor for ESE, which did not support one of the study’s hypotheses. This finding is consistent with previous ESE studies conducted in Spain^23^ and India^22^ but contradictory to a study that used a US sample in which adolescent boys reported higher ESE than girls.^8^ The cultural differences in the upbringing of boys and girls might influence how they perceive and express emotions. Boys might be taught to prioritize stoicism and emotional restraint, which could impact their self-perceived emotional competence.^46^ More research that focuses on elementary school-aged children from various cultural backgrounds is needed to assess whether or not the effects of gender differences on ESE are evident only during certain developmental stages (i.e., transition from middle to high school).

### The Relationship Between Gender and Grade Level With Life Satisfaction

The majority of the children in this sample reported intermediate to high satisfaction in their current lives, with boys more likely to report higher SLSS scores than girls. This finding contrasted with the hypothesized outcome and differed from an earlier study by Huebner,^34^ which found no gender differences regarding reported life satisfaction. Yet this finding is consistent with Aymerich and Casas’ study^36^ that incorporated the use of the Present Overall Life Satisfaction in a sample of high school students. A Russian study noted that, although gender accounted for variances in SLSS scores (girls’ ratings were higher than boys’), the effect size was very small.^42^ These researchers concluded that child personality characteristics, parental stress, parent supervision, and family income are sources of variances in primary school children’s life satisfaction.^42^ Because none of these factors were investigated in this study, further scholarly work should be undertaken to incorporate parent-reported measures and sociodemographic variables to explore the determinants of children’s subjective well-being.

### Limitations

This study was limited to a small sample recruited from an elementary school in one geographic location in the US. Mexican Spanish versions of the SEQ-C and SLSS were not available or validated at the time of the study. Despite translation support, the children’s ratings on both measures might not reflect true scores. Like many other research studies, the reliance on self-reported measures introduces the possibility of response biases that could impact the validity of the study’s conclusions. The combined use of English and Mexican Spanish versions of both instruments in this US-Mexico border community may increase data reliability and validity. Moreover, the study’s cross-sectional design limits its ability to establish causal relationships between emotional self-efficacy and life satisfaction. The use of the convenience sampling method might have introduced potential biases that limited the generalizability of the study results to other populations or contexts.

### Implications for School-Based Practice and Research

The incorporation of self-efficacy and life satisfaction measures into the routine practice of universal screening for mental health in elementary schools is recommended. To promote physical and mental health, the Centers for Disease Control and Prevention recommended the promotion of social, emotional, and behavioral learning through five areas of focus: (1) self-management, (2) responsible decision-making, (3) relationship skills, (4) social awareness, and (5) self-awareness.^47,48^ Increasing ESE may exert a benefit in later life as higher life satisfaction is linked to lower anxiety, depression, and behavior problems, as well as higher educational achievement in reading and math in high school.^49^ Accordingly, school-based intervention programs should be better prepared for students who report low ESE and low life satisfaction. Addressing ESE issues as early as elementary school would enhance students’ life satisfaction as well as prepare them to deal with increasingly complex academic and interpersonal challenges in later childhood. To improve mental health literacy, schools can educate their students via mental health curricula that reduce stigma, improve attitudes/knowledge, and incorporate interactive lessons, in-class group activities, and homework assignments.^50^ Given that mindfulness has been linked to increased life satisfaction among school-age children,^51^ schools can also explore the practice of mindfulness to help their students better manage stress and difficult emotions.^52^

Educators and caregivers of students play a significant role in structuring positive school and home environments that can benefit students’ ESE development. Bandura’s self-efficacy model^5^ suggests that each stressful event serves as a learning opportunity for the student. Therefore, adults can model appropriate behaviors to help children manage stress and negative emotions, provide constructive feedback to students about their social-emotional capabilities during and after stressful events, and carry out healthy practices of eating, exercising, and sleeping with students. Equally important to achieving student’s optimal social-emotional learning, solid home-school relationships are needed.^53,54^ Some useful strategies for educators comprise but are not limited to understanding and supporting diverse family cultures and perspectives on socio-emotional health, building on emotional learning opportunities families already provide at home, ensuring reciprocal and prompt communications with caregivers, and actively engaging families in their children’s social-emotional learning in school.^55^

We recommend future research to partake lessons learned from this study including testing for the reliability and validity of Mexican Spanish versions of the SEQ-C and SLSS, replicating the current study to include more children with Hispanic backgrounds, expanding this study to a longitudinal study, and developing and testing a bilingual program that targets the ESE and subjective well-being of children and families with Hispanic backgrounds. Moreover, researchers can incorporate other influential factors affecting emotional self-efficacy and life satisfaction, such as an individual’s history of effectively coping with stressful situations and their level of contentment with parent-child relationships or family dynamics.

## Conclusion

As part of a larger intervention research project, this study included a predominantly Hispanic elementary school children sample to examine the relationships between ESE and life satisfaction and whether a child’s gender and grade level were significant predictors for these 2 variables. The study findings contribute to positive psychology literature as well as offer valuable insights for school-based mental health practice and research.

## Supplemental Material

sj-docx-1-gph-10.1177_2333794X241286719 – Supplemental material for Perceived Emotional Self-Efficacy and Life Satisfaction of Elementary School Children on the US-Mexico Border Supplemental material, sj-docx-1-gph-10.1177_2333794X241286719 for Perceived Emotional Self-Efficacy and Life Satisfaction of Elementary School Children on the US-Mexico Border by Mei-Ling Lin and Yok-Fong Paat in Global Pediatric Health

sj-docx-2-gph-10.1177_2333794X241286719 – Supplemental material for Perceived Emotional Self-Efficacy and Life Satisfaction of Elementary School Children on the US-Mexico Border Supplemental material, sj-docx-2-gph-10.1177_2333794X241286719 for Perceived Emotional Self-Efficacy and Life Satisfaction of Elementary School Children on the US-Mexico Border by Mei-Ling Lin and Yok-Fong Paat in Global Pediatric Health

## References

[1] National Research Council (US) Panel to Review the Status of Basic Research on School-Age Children. Chapter 1: Introduction. In: Andrew Collins W , ed. Development During Middle Childhood: The Years From Six to Twelve. National Academies Press; 1984.1-23. 25032422

[2] Bright Futures: Guidelines for Health Supervision of Infants, Children, and Adolescents. In: Hagan J. F. Jr. Shaw J. S. Duncan P. M . eds. American Academy of Pediatrics; 2017.

[3] Savi Çakar F . The relationship between the self-efficacy and life satisfaction of young adults. Int Educ Stud. 2012;5(6):123-130. doi:10.5539/ies.v5n6p123

[4] Demirtaş AS. Cognitive flexibility and mental well-being in Turkish adolescents: the mediating role of academic, social and emotional self-efficacy. An de Psicol. 2019;36(1):111-121. doi:10.6018/analesps.336681

[5] Bandura A. Self-Efficacy: The Exercise of Control. W. H. Freeman; 1997.

[6] Yap ST Baharudin R. The relationship between adolescents’ perceived parental involvement, self-efficacy beliefs, and subjective well-being: A multiple mediator model. Soc Indic Res. 2016;126(1):257-278.

[7] Morgan GB Wells KE Andretta JR McKay MT. Temporal attitudes profile transition among adolescents: a longitudinal examination using mover-stayer latent transition analysis. Psychol Assess. 2017;29(7):890-901. doi:10.1037/pas0000383 27599217

[8] Suldo SM Shaffer EJ. Evaluation of the self-efficacy questionnaire for children in two samples of American adolescents. J Psychoeduc Assess. 2007;25(4):341-355.

[9] Di Giunta L Iselin AR Lansford JE , et al Parents’ and early adolescents’ self-efficacy about anger regulation and early adolescents’ internalizing and externalizing problems: a longitudinal study in three countries. J Adolesc. 2018;64:124-135. doi:10.1016/j.adolescence.2018.01.009 29454294 PMC5920650

[10] Dou K Wang Y-J Bin JL Liu YZ. Core self-evaluation, regulatory emotional self-efficacy, and depressive symptoms: testing two mediation models. Soc Behav Pers. 2016;44(3):391-399. doi:10.2224/sbp.2016.44.3.391

[11] Kim Y Kim K Lee S. Testing the self-efficacy questionnaire with Korean children in institutionalized care. Res Soc Work Pract. 2017;27(6):734-742. doi:10.1177/1049731515606219

[12] Muris P. A brief questionnaire for measuring self-efficacy in youths. J Psychopathol Behav Assess. 2001;23(1):145-149.

[13] Mathews BL Koehn AJ Abtahi MM Kerns KA. Emotional competence and anxiety in childhood and adolescence: a meta-analytic review. Clin Child Fam Psychol Rev. 2016;19(2):162-184. doi:10.1007/s10567-016-0204-3 27072682

[14] Muris P. Relationships between self-efficacy and symptoms of anxiety disorders and depression in a normal adolescent sample. Pers Individ Dif. 2002;32(2):337-348.

[15] Zullig KJ Matthews-Ewald MR Valois RF. Weight perceptions, disordered eating behaviors, and emotional self-efficacy among high school adolescents. Eat Behav. 2016;21:1-6. doi:10.1016/j.eatbeh.2015.11.007 26697720

[16] Kokkinos CM Panagopoulou P Tsolakidou I Tzeliou E. Coping with bullying and victimisation among preadolescents: the moderating effects of self-efficacy. Emot Behav Diffic. 2015;20(2):205-222. doi:10.1080/13632752.2014.955677

[17] Valois RF Zullig KJ Hunter AA. Association between adolescent suicide ideation, suicide attempts and emotional self-efficacy. J Child Fam Stud. 2015;24(2):237-248. doi:10.1007/s10826-013-9829-8

[18] Wigelsworth M Qualter P Humphrey N. Emotional self-efficacy, conduct problems, and academic attainment: developmental cascade effects in early adolescence. Eur J Dev Psychol. 2017;14(2):172-189. doi:10.1080/17405629.2016.1180971

[19] Nowland R Qualter P. Influence of social anxiety and emotional self-efficacy on pre-transition concerns, social threat sensitivity, and social adaptation to secondary school. Br J Educ Psychol. 2020;90(1):227-244. doi:10.1111/bjep.12276 30891736

[20] Phan HP Ngu BH. Sources of self-efficacy in academic contexts: a longitudinal perspective. Sch Psychol Q. 2016;31(4):548-564. doi:10.1037/spq0000151 26985966

[21] Verlenden JV Pampati S Rasberry CN , et al Association of children’s mode of school instruction with child and parent experiences and well-being during the COVID-19 pandemic - COVID Experiences Survey, United States, October 8-November 13, 2020. MMWR Morb Mortal Wkly Rep. 2021;70(11):369-376. 33735164 10.15585/mmwr.mm7011a1 PMC7976614

[22] Kaur A Singh A. A study of self-efficacy in terms of gender differences among school students. Indian J Heal Well. 2017;8(10):1194-1198.

[23] Salavera C Usán P Jarie L. Emotional intelligence and social skills on self-efficacy in secondary education students. Are there gender differences? J Adolesc. 2017;60:39-46. doi:10.1016/j.adolescence.2017.07.009 28750267

[24] Huebner ES. Initial development of the Student’s Life Satisfaction Scale. Sch Psychol Int. 1991;12(3):231-240.

[25] Pavot W Diener E. The satisfaction with life scale and the emerging construct of life satisfaction. J Posit Psychol. 2008;3(2):137-152. doi:10.1080/17439760701756946

[26] Kim ES Delaney SW Tay L Chen Y Diener ED Vanderweele TJ Life satisfaction and subsequent physical, behavioral, and psychosocial health in older adults. Milbank Q. 2021;99(1):209-239. 33528047 10.1111/1468-0009.12497 PMC7984669

[27] Veenhoven R. Why studies in the effect of positive psychological interventions should use life-satisfaction as an outcome. Front Psychol. 2021;12:758623. 34899500 10.3389/fpsyg.2021.758623 PMC8654934

[28] Proctor C Alex Linley P Maltby J. Youth life satisfaction measures: a review. J Posit Psychol. 2009;4(2):128-144. doi:10.1080/17439760802650816

[29] Seligson JL Huebner ES Valois RF. An investigation of a brief life satisfaction scale with elementary school children. Soc Indic Res. 2005;73(3):355-374.

[30] Migliorini L Tassara T Rania N. A study of subjective well-being and life satisfaction in Italy: how are children doing at 8 years of age? Child Indic Res. 2019;12(1):49-69.

[31] Jovanović V Rudnev M Arslan G , et al. The Satisfaction with Life Scale in Adolescent Samples: Measurement Invariance across 24 Countries and Regions, Age, and Gender. Appl Res Qual Life. 2022;17(4):2139-2161. doi:10.1007/s11482-021-10024-w 35096193 PMC8784202

[32] Jiang X Fang L Stith BR Liu R-D Huebner ES. A cross-cultural evaluation of the students’ life satisfaction scale in Chinese and American adolescents. Curr Psychol. 2021;40(5):2552-2560.

[33] Huebner ES Ostafińska-Molik B Gaweł A. Students’ Life Satisfaction Scale (SLSS): psychometric properties with a sample of Polish adolescents. Appl Res Qual Life. 2022;17(4):2191-2209.

[34] Huebner ES. Correlates of life satisfaction in children. Sch Psychol Q. 1991;6(2):103-111.

[35] Lewis AD Huebner ES Malone PS Valois RF. Life satisfaction and student engagement in adolescents. J Youth Adolesc. 2011;40(3):249-262. doi:10.1007/s10964-010-9517-6 20204687

[36] Aymerich M Casas F. A contextualized measure of overall life satisfaction among adolescents: differences by gender. Child Indic Res. 2020;13(6):2241-2260.

[37] Terry T Scott Huebner E. The relationship between self-concept and life satisfaction in children. Soc Indic Res. 1995;35(1):39-52.

[38] Danielsen AG Samdal O Hetland J Wold B. School-related social support and students’ perceived life satisfaction. J Educ Res. 2009;102(4):303-320. doi:10.3200/joer.102.4.303-320

[39] Chen X Cai Z He J Fan X. Gender differences in life satisfaction among children and adolescents: a meta-analysis. J Happiness Stud. 2020;21(6):2279-2307.

[40] Lin ML Fierro C Medrano C Arroyos D Medrano G . Addressing mental health needs of elementary school children through university-community collaboration. AJOT Special Interest Section Quarterly Practice Connections. 2020; 5(3):20-22.

[41] Minter A Pritzker S. Measuring adolescent social and academic self-efficacy: cross-ethnic validity of the SEQ-C. Res Soc Work Pract. 2017;27(7):818-826.

[42] Huebner ES. The Students’ Life Satisfaction Scale: an assessment of psychometric properties with black and White elementary school students. Soc Indic Res. 1995;34(3):315-323.

[43] Leto IV Petrenko EN Slobodskaya HR. Life satisfaction in Russian primary schoolchildren: links with personality and family environment. J Happiness Stud. 2019;20(6):1893-1912.

[44] Wingate EJ Suldo SM Peterson RKS . Monitoring and fostering elementary school students’ life satisfaction: a case study. J Appl Sch Psychol. 2018;34(2):180-200.

[45] McDevitt T Ormrod J Cupit G Chandler M Aloa V. Child Development and Education. Pearson Education; 2010.

[46] Mahalik JR Burns SM Syzdek M. Masculinity and perceived normative health behaviors as predictors of men’s health behaviors. Soc Sci Med. 2007;64(11):2201-2209. doi:10.1016/j.socscimed.2007.02.035 17383784

[47] Division of Adolescent and School Health NCfCDPaHP. Promote social, emotional, and behavioral learning. Updated December 6, 2023. Accessed June 27, 2024. https://www.cdc.gov/healthyyouth/mental-health-action-guide/promote-social-emotional-and-behavioral-learning.html

[48] Division of Adolescent and School Health NCfCDPaHP. Supporting social and emotional learning with student-led clubs. Updated July 2, 2021. Accessed June 27, 2024. https://www.cdc.gov/healthyyouth/stories/mh_orange-county.htm

[49] State TM Kern L. Life satisfaction among high school students with social, emotional, and behavioral problems. J Posit Behav Interv. 2017;19(4):205-215. doi:10.1177/1098300717714573

[50] Division of Adolescent and School Health NCfCDPaHP. Increase students’ mental health literacy. Updated December 6, 2023. Accessed June 27, 2024. https://www.cdc.gov/healthyyouth/mental-health-action-guide/increase-students-mental-health-literacy.html

[51] Amundsen R Riby LM Hamilton C Hope M McGann D. Mindfulness in primary school children as a route to enhanced life satisfaction, positive outlook and effective emotion regulation. BMC Psychol. 2020;8(1):71. doi:10.1186/s40359-020-00428-y 32641161 PMC7341670

[52] Division of Adolescent and School Health NCfCDPaHP. Promote mindfulness. Updated December 6, 2023. Accessed June 27, 2024. https://www.cdc.gov/healthyyouth/mental-health-action-guide/promote-mindfulness.html

[53] Bachman HF Cunningham PD Boone BJ. Collaborating with families for innovative school mental health. Educ Sci. 2024;14(3):336.

[54] Conroy K Hong N Poznanski B , et al Harnessing home-school partnerships and school consultation to support youth with anxiety. Cogn Behav Pract. 2022;29(2):381-399. doi:10.1016/j.cbpra.2021.02.007 35812004 PMC9267952

[55] Couchenour D Chrisman K. Families, Schools and Communities: Together for Young Children. Cengage Learning; 2013.

