# Supplementary material for: Perceived Emotional Self-Efficacy and Life Satisfaction of Elementary School Children on the US-Mexico Border
Source: Glob Pediatr Health. 2024 Sep 27;11:2333794X241286719. doi: 10.1177/2333794X241286719 (PMC11456195; doi:10.1177/2333794X241286719)
Supplement: sj-docx-1-gph-10.1177_2333794X241286719 – Supplemental material for Perceived Emotional Self-Efficacy and Life Satisfaction of Elementary School Children on the US-Mexico Border [file sj-docx-1-gph-10.1177_2333794X241286719.docx]

**Self-Efficacy Questionnaire for Children (SEQ-C)- Pre**

| During the past week, | | 1  Not at all | 2 | 3  Fairly well | 4 | 5  Very well |
| --- | --- | --- | --- | --- | --- | --- |
| 1. | How well do you succeed in cheering yourself up when an unpleasant event has happened? | 0 | 0 | 0 | 0 | 0 |
| 2. | How well do you succeed in becoming calm again when you are very scared? | 0 | 0 | 0 | 0 | 0 |
| 3. | How well can you prevent to become too nervous? | 0 | 0 | 0 | 0 | 0 |
| 4. | How well can you control your negative feelings? | 0 | 0 | 0 | 0 | 0 |
| 5. | How well can you give yourself a pep-talk when you feel low? | 0 | 0 | 0 | 0 | 0 |
| 6. | How well can you tell a friend that you don’t feel well? | 0 | 0 | 0 | 0 | 0 |
| 7. | How well do you succeed in suppressing unpleasant thoughts? | 0 | 0 | 0 | 0 | 0 |
| 8. | How well do you succeed in not worrying about things that might happen? | 0 | 0 | 0 | 0 | 0 |
