# Supplementary material for: Perceived Emotional Self-Efficacy and Life Satisfaction of Elementary School Children on the US-Mexico Border
Source: Glob Pediatr Health. 2024 Sep 27;11:2333794X241286719. doi: 10.1177/2333794X241286719 (PMC11456195; doi:10.1177/2333794X241286719)
Supplement: sj-docx-2-gph-10.1177_2333794X241286719 – Supplemental material for Perceived Emotional Self-Efficacy and Life Satisfaction of Elementary School Children on the US-Mexico Border [file sj-docx-2-gph-10.1177_2333794X241286719.docx]

**Students’ Life Satisfaction Scale**

**(Huebner, 1991)**

**Directions:** We would like to know what thoughts about life you have had during the past several weeks. Think about how you spend each day and night and then think about how your life has been during most of this time. Here are some questions that ask you to indicate your satisfaction with your overall life. Circle the words next to each statement that indicate the extent to which you agree or disagree with each statement.

For example, if you Strongly Agree with the statement “Life is great,” you would circle those words on the following sample item;

| **Life is great.** | Strongly Disagree | Moderately Disagree | Mildly Disagree | Mildly Agree | Moderately Agree | Strongly Agree |
| --- | --- | --- | --- | --- | --- | --- |

It is important to know what you REALLY think, so please answer the questions the way you really think, not how you should think. This is NOT a test. There are NO right or wrong answers.

| 1. **My life is going well.** | Strongly Disagree | Moderately Disagree | Mildly Disagree | Mildly Agree | Moderately Agree | Strongly Agree |
| --- | --- | --- | --- | --- | --- | --- |
| 1. **My life is just right.** | Strongly Disagree | Moderately Disagree | Mildly Disagree | Mildly Agree | Moderately Agree | Strongly Agree |
| 1. **I would like to change many things in my life.** | Strongly Disagree | Moderately Disagree | Mildly Disagree | Mildly Agree | Moderately Agree | Strongly Agree |
| 1. **I wish I had a different kind of life.** | Strongly Disagree | Moderately Disagree | Mildly Disagree | Mildly Agree | Moderately Agree | Strongly Agree |
| 1. **I have a good life.** | Strongly Disagree | Moderately Disagree | Mildly Disagree | Mildly Agree | Moderately Agree | Strongly Agree |
| 1. **I have what I want in life.** | Strongly Disagree | Moderately Disagree | Mildly Disagree | Mildly Agree | Moderately Agree | Strongly Agree |
| 1. **My life is better than most.** | Strongly Disagree | Moderately Disagree | Mildly Disagree | Mildly Agree | Moderately Agree | Strongly Agree |

Huebner, E. S. (1991). Initial development of the Students’ Life Satisfaction Scale. *School Psychology International, 12,* 231-243.
